# The association between ROS1 rearrangement and risk of thromboembolic events in patients with advanced non-small cell lung cancer: a multicenter study in China

**DOI:** 10.1186/s12959-022-00417-8

**Published:** 2022-09-27

**Authors:** Jiawen Yi, Huang Chen, Jie Li, Xingran Jiang, Yan Xu, Mengzhao Wang, Zheng Wang, Zhenguo Zhai, Yanhong Ren, Yuhui Zhang

**Affiliations:** 1 Department of Respiratory and Critical Care Medicine, Beijing Chao-Yang Hospital, Capital Medical University, Beijing Institute of Respiratory Medicine, Beijing, China; 2 Department of Pathology, China-Japan Friendship Hospital, Beijing, China; 3 Department of Pathology, Beijing Chao-Yang Hospital, Capital Medical University, Beijing, China; 4 Department of Respiratory and Critical Care Medicine, Peking Union Medical College Hospital, Peking Union Medical College and Chinese Academy of Medical Sciences, Beijing, China; 5 Department of Pathology, Beijing Hospital, National Center of Gerontology, Institute of Geriatric Medicine, Chinese Academy of Medical Sciences, Beijing, China; 6 Department of Pulmonary and Critical Care Medicine, Center of Respiratory Medicine, China-Japan Friendship Hospital, Beijing, China

**Keywords:** Non-small cell lung cancer, Advanced, Thromboembolic events, ROS1 rearrangement

## Abstract

**Background:**

According to several studies, ROS1 rearrangement is associated with thrombotic risk in non-small cell lung cancer (NSCLC). However, there is no clear understanding of the predictors and prognostic impact of thromboembolic events (TEEs) in patients with advanced ROS1 rearrangement NSCLC.

**Methods:**

A total of 47 newly diagnosed advanced NSCLC patients with ROS1 rearrangement from four Chinese hospitals were retrospectively included and were evaluated for TEEs incidence, characteristics, predictors, as well as response to therapies and overall survival (OS).

**Results:**

Of the 47 enrolled patients, 23.4% (*n* = 11) patients developed TEEs. Among them, 7 of 11 patients (64%) developed pulmonary embolism (PE), and 5 patients (45%) experienced recurrent TEEs. In multivariate analysis, D-dimer was associated with the occurrence of TEEs in ROS1 rearranged NSCLC (HR 1.16, 95% CI 1.08–1.23, *P* < 0.001). Median progression-free survival (PFS) after first-line ROS1 tyrosine kinase inhibitors (TKIs) therapy was significantly longer in patients without TEEs than in those developing TEEs (26 months vs. 12 months, *P* = 0.0383). Furthermore, patients with TEEs had a shorter OS period than those without TEEs (29.8 months vs. not estimable, *P* = 0.0647).

**Conclusion:**

The results of this multicenter study indicated that advanced NSCLC patients with ROS1 rearrangement were more likely to experience PE and TEEs recurrence. And patients with TEEs tended to have a worse prognosis. Furthermore, an elevated D-dimer level suggested a hypercoagulable state in NSCLC patients with ROS1 rearrangement.

## Background

Thromboembolic events (TEEs) are common complications in patients with non-small cell lung cancer (NSCLC), with an incidence rate between 10 and 15% [1, 2]. Considerable evidence indicates that thrombotic complications are associated with a worse prognosis and are the second most frequent cause of death in cancer patients [3–5].

Molecular testing has become a standard procedure among patients with NSCLC. Previous research proved that specific mutations including epidermal growth factor receptor (EGFR) mutations and anaplastic lymphoma kinase (ALK) rearrangement played a role in thrombotic risk in NSCLC [6, 7].

Also, ROS1 rearrangement exhibited biological homology with ALK rearrangement [8], and a number of case-control and cohort studies demonstrated that ROS1 rearrangement was associated with TEEs in NSCLCs as well [9–12]. The prevalence of ROS1 rearrangement is reported to be less than 2% in NSCLC among Caucasian populations and is slightly higher in the East Asian populations with a prevalence of 2–3% [13]. Currently, the treatment status in China differs from that in developed countries. Since crizotinib was approved in China for ROS1-rearranged NSCLC in 2017 [14], chemotherapy was mainly adopted before 2017 among these patients. Meanwhile, little is known about the clinical relevance and characteristics of ROS1 rearrangement in Chinese NSCLC patients. The primary objective of the present multicenter study was to investigate the incidence and risk factors of TEEs in ROS1 rearrangement with newly diagnosed NSCLC in China. The secondary objective was to explore the treatment response and survival among ROS1 rearrangement NSCLC patients with TEEs and without TEEs.

## Material and methods

### Study populations

The ROS1-rearranged advanced NSCLC cohort included patients attending four Chinese hospitals from March 2015 to March 2021. The retrospective observational cohort included patients who met the following inclusion criteria: histological confirmation of the diagnosis; ROS1 rearrangement determined by fluorescence in situ hybridization (FISH), immunohistochemistry (IHC), reverse transcription-polymerase chain reaction (RT-PCR), or next generation sequencing (NGS). The exclusion criteria were as follows: any surgery, chemotherapy, or radiotherapy within 3 months before diagnosis; insufficient available clinical data and uncooperative follow-up. This study was approved by the central Ethics Committee of Beijing Chao-Yang Hospital, Capital Medical University, Beijing, China (No.2021-KE-443).

### Outcome measurement

In this study, the primary outcome is the occurrence of TEEs, including arterial thromboembolism (ATE) and venous thromboembolism (VTE). The incidence of TEEs was estimated 3 months before diagnosis and 6 months after diagnosis, and the final follow-up date was September 2021.

Routine screening for TEEs was not conducted, and symptomatic or incidental TEEs were confirmed by objective imaging methods. VTE includes deep vein thrombosis (DVT) and pulmonary embolism (PE). Objective evidence for the diagnosis of DVT included venous ultrasound imaging or computed tomography (CT) angiography. And the objective evidence for the diagnosis of PE included CT pulmonary angiogram or ventilation-perfusion scan. ATE was defined as a composite of acute myocardial infarction, peripheral arterial occlusion, and stroke. To confirm the stroke diagnosis, CT or magnetic resonance imaging (MRI) was performed. The diagnosis of peripheral arterial occlusion was confirmed by the use of doppler-sonography, digital subtraction angiography, CT-angiography, and MRI-angiography. The diagnosis of myocardial infarction was confirmed by the use of echocardiography, angiography or cardiac biomarkers.

### Data collection

Patient-related factors (age, sex, performance status, smoking history, and medical records), tumor-related factors (tumor histology, tumor stage, oncogenic status, occurrence time of TEEs, patients’ situation of TEEs and the location of TEEs), treatment-related factors (anti-tumor therapy and anticoagulant therapy) and baseline laboratory variables (complete blood count, serum carcinoembryonic antigen (CEA), lactate dehydrogenase (LDH), C-reactive protein (CRP) and D-dimer) were extracted from the patients’ electronic clinical records and collected from patients themselves via telephone follow-up.

In addition, we used the Charlson Comorbidity Index (CCI) to measure the burden of comorbidity. According to the calculated CCI scores, we categorized all patients into three groups: low (no comorbidity): CCI score of 0, medium: CCI score of 1–2, or high: CCI score of 3 or more.

### Statistical analysis

Continuous variables were described by using median and range, and transformed into categorical variables by defining the best cut-offs using the maximum selection test method. Categorical variables were described as frequencies and percentages.

The cumulative incidence of TEEs was estimated using competing risk analysis, treating death as a competing event. And Grey’s test and Fine and Gray competing regression, with death as a competing risk, were conducted to identify the risk factors associated with TEEs. A value of *P* < 0.05 was considered statistically significant.

Progression-free survival (PFS) and overall survival (OS) were estimated by Kaplan-Meier (KM) method and were defined as NSCLC diagnosis to disease progression and death by any cause. The response was reported by the treating clinician according to the Response Evaluation Criteria In Solid Tumors version 1.1 (RECIST 1.1).

R statistical software version 4.1.0 was used to perform the maximum selection test and Fine and Gray competing regression analysis. Grey’s test analyses were performed using NCSS 12. Statistical analysis was performed using SPSS version 21.0. This study was reviewed by a professional epidemiologist.

## Results

### Patient characteristics

A total of 47 patients diagnosed with ROS1-rearranged NSCLC from four hospitals in Northern China were included in the study. The 47 patients had a mean age of 54.38 years (SD, 11.57) at diagnosis; 57% (27 of 47) of patients were female. A majority of the patients had a performance status (PS) of 0 to 1 (36 of 47, 77%), and 68% (32 of 47) of the patients were non-smokers. Our analysis of the medical records of the patients revealed that 25% (12 of 47) of the patients had a low CCI score, 53% (25 of 47) had a medium CCI score, and 21% (10 of 47) had a high CCI score.

And we also evaluated the tumor characteristics of the cohort. The population consisted of 47 patients with adenocarcinoma (46 of 47, 98%) and 1 patient with adenosquamous carcinoma (1 of 47, 2%). All patients had advanced disease stages at diagnosis, of which 70% (33 of 47) of patients had at least 2 sites of metastases, and 30% (14 of 47) of patients had 3 and more sites of metastases. Furthermore, 39 patients (39 of 47, 85%) had distant metastases; 6 had brain metastases, 22 had pleural metastases, and 13 had bone metastases (Table 1).

**Table 1:** Baseline demographic and clinical characteristics of the study population (*N* = 47)

Characteristics	
**Mean age at diagnosis (SD), y**	54.38 (11.57)
**Sex** (%)	
Male	20 (43)
Female	27 (57)
**Performance status (%)**	
<2	36 (77)
≥ 2	11 (23)
**Smoking history (%)**	
Current and former	15 (32)
Never	32 (68)
**Charlson Comorbidity Index (%)**	
0	12 (25)
1–2	25 (53)
≥ 3	10 (21)
**Histology (%)**	
Adenocarcinoma	46 (98)
Adenosquamous carcinoma	1 (2)
**Stage at diagnosis**	
IIIB	7 (15)
IV	40 (85)
**Number of metastatic sites (%)**	
1–2	33 (70)
≥ 3	14 (30)
**Metastatic site Brain (%)**	
No	41 (87)
Yes	6 (15)
**Liver (%)**	
No	45 (96)
Yes	2 (4)
**Lung (%)**	
No	35 (74)
Yes	12 (26)
**Adrenal (%)**	
No	43 (91)
Yes	4 (9)
**Soft tissue (%)**	
No	45 (96)
Yes	2 (4)
**Bone (%)**	
No	34 (72)
Yes	13 (28)
**Pleural (%)**	
No	25 (53)
Yes	22 (47)
**Lymph node (%)**	
No	13 (28)
Yes	34 (72)
**All lines therapy (%)**	
Chemotherapy	19 (26)
ROS1TKIs	37 (51)
Others	16 (22)
**First-line therapy (%)**	
First-line ROS1 TKIs	24 (51)
Chemotherapy	13 (28)
Others	10 (21)
**Ros-1 TKIs (%)**	
Ros-1 TKIs at first-line therapy	24 (65)
Ros-1 TKIs at any line therapy	13 (35)
**Baseline laboratory values (Means, SD)**	
Leukocyte count	8.26 (4.04)
Neutrophil count	5.98 (3.83)
Hemoglobin	135.67 (16.56)
Platelet count	281.78 (76.04)
Carcinoembryonic antigen (CEA)	15.34 (33.44)
Lactate dehydrogenase (LDH)	238.56 (109.37)
C-reactive protein (CRP)	13.67 (18.00)
D-dimer	3.97 (6.41)

*Abbreviations*: *SD* Standard deviation, *TKIs* Tyrosine kinase inhibitors, *CEA* Carcinoembryonic antigen, *LDH* Lactate dehydrogenase, *CRP* C-reactive protein

### Development and recurrence of thromboembolic events

Of the NSCLC patients with ROS1 rearrangement, 11 (11 of 47, 23.4%) of 47 patients developed TEEs within 3 months prior to and 6 months after diagnosis. TEEs consisted of 9 VTE events (9 of 11, 72%) and 2 ATE combined VTE events (2 of 11, 28%). As for the VTE group, 4 patients had only DVT or only PE (4 of 9, 44%), while 5 had both DVT and PE (5 of 9, 56%). Meanwhile, among the entire TEEs group, 2 patients (2 of 11, 18%) developed both ATE and VTE. Overall, 7 patients (7 of 11, 64%) developed PE during follow-up. The majority of the patients developed TEEs symptomatically (10 of 11, 91%).

In the investigation of the TEEs occurrence, there were 5 ambulatory patients (5 of 11, 45%) and 6 hospitalized patients (6 of 11, 55%). Most of the TEEs (7 of 11, 64%) developed before treatment, and TEEs occurred at disease progression in 18% of cases. There were 4 patients (4 of 11, 36%) who developed TEEs during anti-tumor therapy. Almost half of the patients (5 of 11, 45%) suffered recurrent TEEs, and all the relapses happened under therapeutic anticoagulation, of which 4 patients took low molecular weight heparin (LMWH). Among the patients with TEEs recurrence, 4 patients (4 of 11, 36%) received chemotherapy while 6 patients (6 of 11, 55%) received ROS1 TKIs. More than half of the patients (3 of 4, 75%) in the chemotherapy group presented TEEs relapse and the recurrence rate in the ROS1 TKIs group was 33% (1 of 3), which showed a significant difference between these two therapies (Pearson Chi-Square 10.0, *P* = 0.005).

**Fig. 1:**
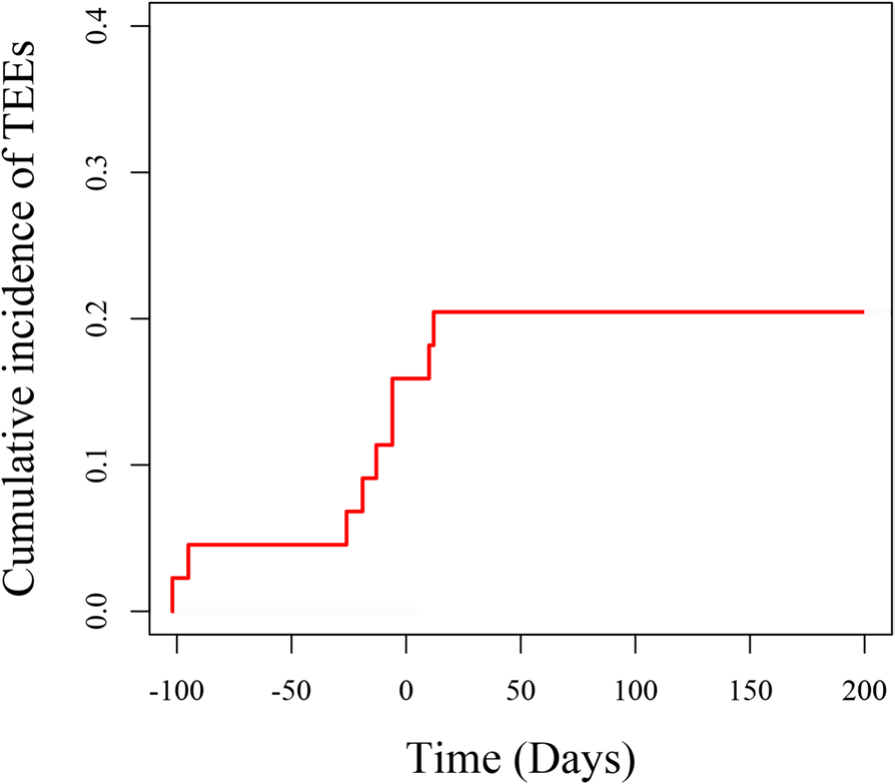
The cumulative incidence of TEEs in ROS1 rearranged NSCLC analyzed by competing risk analysis

The clinical characteristics of TEEs were listed in Table 2, and the cumulative incidence of TEEs by Fine and Gray competing-risks regression with death as a competing risk was illustrated in Fig. 1.

**Table 2:** Incidence and characteristics of TEEs in ROS1 rearranged NSCLC

Characteristics	Number of patients (%)
**Total**	11 (11/47, 23.4%)
**Type of thromboembolic disease**	
Venous	9 (81)
Arterial	0 (0)
Both venous and arterial	2 (19)
**Patients’ situation**	
Ambulatory	5 (45)
Hospitalized	6 (55)
**Time of occurrence**	
Before starting treatment	7 (63)
During treatment	4 (37)
**Situation of occurrence of TEEs**	
Symptomatic	10 (91)
Incidental	1 (9)
**Time of occurrence according to the clinical situation of the disease**	
Untreated	7 (63)
During platinum-based chemotherapy	2 (28)
During immune checkpoint therapy	1 (9)
During ROS1 TKIs treatment	1 (9)
**Time of occurrence according to the clinical situation of the disease**	
Untreated	7 (63)
Partial response	0 (0)
Stable disease	2 (18)
Progressive disease	2 (18)
**Location of TEEs**	
Only VTE	
a) Only deep vein thrombosis (DVT)	3 (27)
Lower extremity DVT	2 (67)
Upper extremity DVT	1 (33)
Lower extremity and upper extremity DVT	0 (0)
b) Only pulmonary embolism (PE)	1 (9)
c) DVT and PE combined	5 (45)
Lower extremity DVT and PE	3 (60)
Upper extremity DVT and PE	1 (20)
Lower extremity, upper extremity DVT and PE	1 (20)
Both ATE and VTE	2 (28)
a) Stroke and DVT	1 (50)
b) Stroke and PE	1 (50)
**Recurrent TEEs**	
Yes	5 (45)
under therapeutic anticoagulation	5 (100)
not under therapeutic anticoagulation	0 (0)
No	6 (55)
**First-line anti-tumor treatment in patients with TEEs recurrence**	
During chemotherapy	3 (3/4, 75%)
During ROS1 TKIs treatment	2 (2/6, 33%)
**Anticoagulation therapy**	
Low molecular weight heparin	8 (73)
Novel oral anticoagulant	3 (27)

*Abbreviation*: *TEEs* Thromboembolic events, *VTE* Venous thromboembolism, *ATE* Arterial thromboembolism, *DVT* Deep vein thrombosis, *PE* Pulmonary embolism

### Risk factors of TEEs in ROS1 rearranged NSCLC

For numeric variables in this cohort, laboratory parameters were categorized based on the best cut-offs determined by the maximum selection test. Prognostic factors and biomarkers were identified in univariate analysis. Additionally, the following variables were significantly associated with symptomatic or incidental TEEs occurrence in ROS1 rearranged NSCLC: (1) LDH > 312 U/L (*P* = 0.003); (2) D-dimer > 7.8μg/ml (*P* = 0.02) (Table 3).

**Table 3:** Factors associated with increased risk of TEEs in patients with ROS1 rearranged NSCLC

Variables	Number of patients (%)		Univariate Analysis		Multivariate Analysis	
	With TEEs (***n*** = 11)	Without TEEs (***n*** = 36)	χ2	***P*** value	sHR (95% CI)	***P*** value
**Age**			1.9528	0.162		
< 60	8(73)	23(64)				
≥ 60	3(27)	13(36)				
**Sex**			1.7999	0.180		
Male	6(55)	14(39)				
Female	5(45)	22(61)				
**Body-mass index**			1.0295	0.310		
< 23 kg/m2	4(36)	19(53)				
≥ 23 kg/m2	7(64)	17(47)				
**Performance status**			0.6107	0.435		
0–1	3(27)	25(69)				
≥ 2	8(73)	11(31)				
**Charlson Comorbidity Index**			1.8064	0.405		
0	4(36)	8(22)				
1–2	5(45)	20(56)				
≥ 3	2(18)	8(22)				
**Smoke History**			0.4280	0.513		
Current and Former	4(36)	12(33)				
Never	7(64)	24(67)				
**Number of metastatic sites**			0.7639	0.382		
1–2	7(64)	25(69)				
≥ 3	4(36)	11(31)				
**Metastatic sites**						
Brain	0(0)	6(17)	0.8833	0.347		
Liver	1(10)	1(3)	0.1264	0.722		
Lung	3(30)	9(25)	0.0049	0.944		
Adrenal	1(10)	3(8)	0.8671	0.352		
Soft tissue	0(0)	2(6)	0.2604	0.610		
Bone	2(20)	11(31)	1.5584	0.212		
Pleural	6(55)	16(44)	1.2828	0.257		
Lymph node	11(100)	23(64)	1.9578	0.162		
**Baseline laboratory values**						
Leukocyte count>5.61 × 10^9^/L			0.8359	0.361		
Neutrophil count>4.06 × 10^9^/L			1.5768	0.209		
Hemoglobin ≤143 g/L			1.8735	0.171		
Platelet count >220 × 10^9^/L			0.8769	0.349		
CEA>4.52 ng/ml			0.3222	0.570		
LDH>312 U/L			13.5355	< 0.001	1.0 (1.00–1.01)	0.13
CRP>18.9 mg/L			2.2882	0.130	1.0 (0.98–1.03)	0.79
D-dimer>7.8μg/ml			5.0609	0.02	1.16 (1.08–1.24)	< 0.001

*Abbreviations*: *TEEs* Thromboembolic events, *CEA* Carcinoembryonic antigen, *LDH* Lactate dehydrogenase, *CRP* C-reactive protein

In multivariate analysis, we selected the top three strongest predictors in univariate analysis (LDH, D-dimer, and CRP), and incorporated them into multivariate analysis. The results showed that a high level of D-dimer was of significant association with increased thrombotic risk in ROS1 rearranged NSCLC patients (HR 1.16, 95% CI 1.08–1.23, *P* < 0.001) (Table 3).

### Response and survival

According to different treatments, 19 patients (19 of 72, 26%) received chemotherapy, 37 patients (37 of 72, 51%) were treated with ROS1 TKIs, and 16 patients (16 of 72, 22%) received other treatments (such as radiotherapy, palliative surgery, immune checkpoint therapy, and traditional Chinese medicine therapy). And among patients taking ROS1 TKIs at any line, 24 patients (24 of 37, 65%) adopted it as first-line therapy, while 13 patients (13 of 37, 35%) adopted it as second/third-line therapy (Table 1). Of 11 patients with TEEs, 6 patients (6 of 11, 55%) received ROS1 TKIs at any line therapy.

**Fig. 2:**
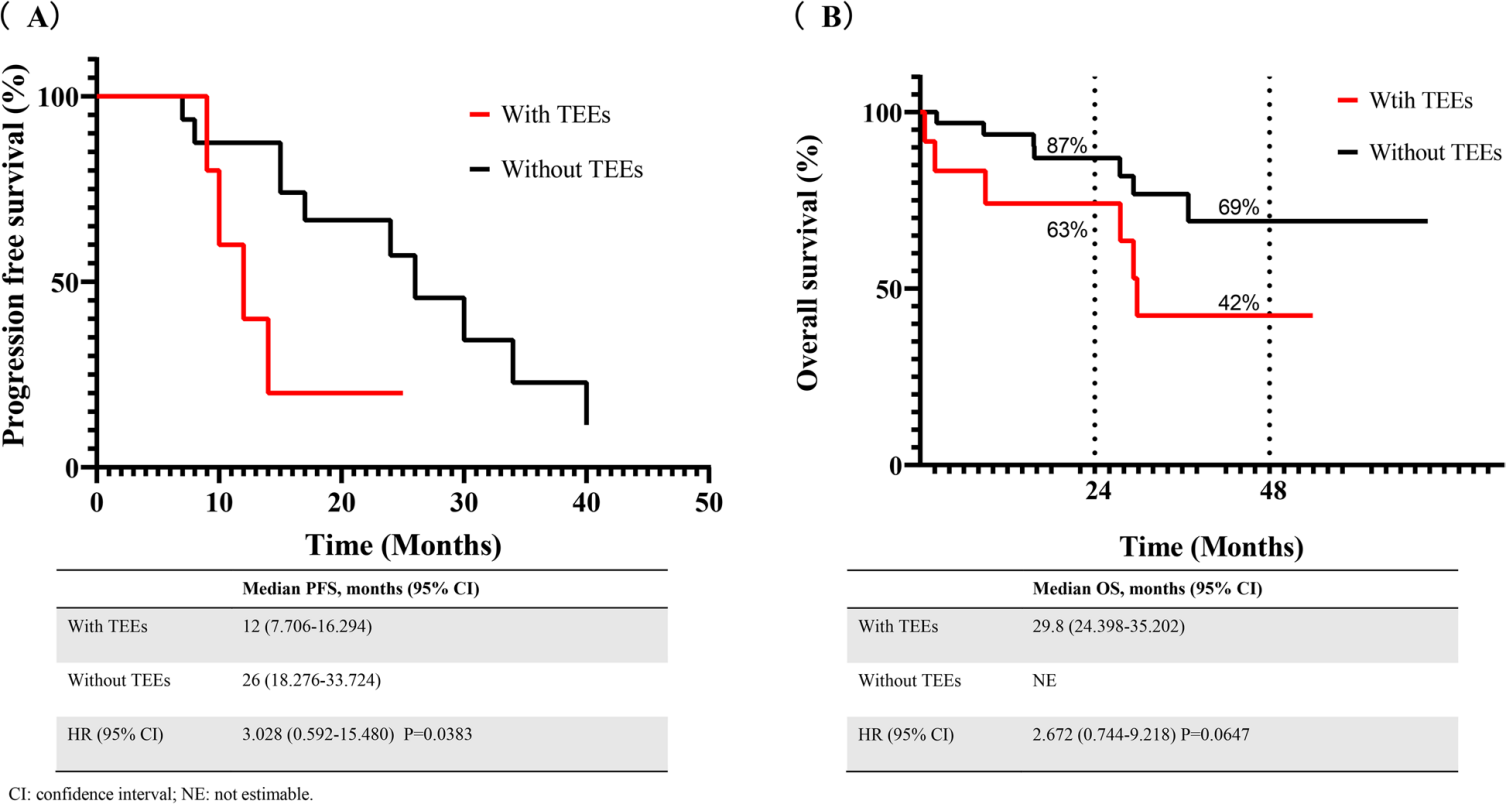
Kaplan-Meier analysis (**A**) Comparison of PFS between ROS1 rearranged NSCLC patients receiving ROS1 TKIs with and without TEEs (*P* = 0.0383). (**B**) Comparison of OS between ROS1 rearranged NSCLC patients with or without TEEs (*P* = 0.0647)

Moreover, at a median follow-up of 28.4 months, we evaluated the association between TEEs, PFS, and OS using KM analysis. In the 37 patients treated with ROS1 TKIs at first-line, patients with TEEs had significantly shorter PFS than patients without TEEs (12 vs. 26 months, *P* = 0.0383) (Fig. 2A). In the 47 ROS1 rearranged NSCLC patients, 5 of 11 (45%) and 8 of 36 (22%) died in the TE and non-TE cohorts. The median OS was 29.8 months with at least one TEE, whereas this was not reached in the group without TEEs (29.8 months vs. not estimable, *P* = 0.0647). According to the KM curve, the OS probability in patients with and without TEEs was 63% vs. 87% at 24 months, and was 42% vs. 69% at 48 months (Fig. 2B).

Additionally, we also evaluated the response to ROS1 TKIs in different lines of therapy among patients with ROS1 rearranged NSCLC. Patients treated with ROS1 TKIs at first-line (*n* = 24) showed a better response to TKIs than those at second/third-line (*n* = 13). The median PFS for the patients receiving ROS1 TKIs at first line treatment was 24 months whereas second/third line treatment was 12 months (24 vs. 12 months, *P* = 0.0446).

## Discussion

Previous studies on ROS1-rearranged NSCLC mostly came from white patients.. Although the prevalence of ROS1-rearrangement was higher in East Asia and the therapy regimens were limited in China, the results of this Chinese multicentre observational study showed that the presence of ROS1 rearranged in NSCLC may increase the risk of TEEs. This study also investigated the risk factors for TEEs among ROS1-rearranged NSCLC, and analyzed the different responses to ROS1 TKIs and OS between TEEs and without TEEs group.

The incidence of TEEs was relatively lower in this population than in patients from previous studies (Table 4). Since ROS1 rearrangement is a rare variation, the sample size of these studies was relatively small. Meanwhile, the observation periods were different in these studies. TEEs were estimated between 3 months prior to and 6 months after the NSCLC diagnosis since the TEEs during this period were more relevant to cancer.

**Table 4:** Prevalence of TEEs in ROS1-rearranged NSCLC in different studies

Study	Country	Study type	Total patients	TEEs observation period	Incidence
Ng et al. [9]	USA, China	Retrospective cohort study	95	Within ±90 days of diagnosis	34.7%
Chiari et al. [10]	Italy	Randomized controlled trial	48	From diagnosis to last follow up or death	41.6%
Alexander et al. [11]	Australia	Retrospective cohort study	42	1-year prior to diagnosis until the last study follow-up	47.6%
Muñoz-Unceta et al. [12]	Spain, Portugal	Retrospective cohort study	58	6 months before diagnosis to last follow up or death	46.6%
Current study	China	Retrospective cohort study	47	3 months before diagnosis to 6 months after diagnosis	23.4%

Notably, more than half of the TEEs cohort experienced PE, many times the incidence (2–4%) in the general cohort [15, 16]. The high incidence of PE indicated that the patients with ROS1 rearranged NSCLC were in a hypercoagulable state, and whether or not to undertake thromboprophylaxis needs careful consideration. Meanwhile, TEEs occurred at time points across the patients’ disease course, including pre and post-treatment and throughout the disease course. In the TEEs cohort, most of the patients developed TEEs before treatment. As in previous studies, suspicion of underlying cancer is frequently raised in patients with TEEs [17].

Furthermore, half of the patients in the TEEs cohort experienced TEEs recurrence, which was 1.8-fold higher than the general cancer populations [18]. And all of them were during anticoagulant treatment. Meanwhile, these patients experienced TEEs relapse within 6 months of their first occurrence, which caused us to focus on anticoagulant treatment in the first 6 months after the first occurrence of TEEs in ROS1-rearranged NSCLC patients. Previous studies showed that chemotherapy was a clinical risk factor for TEEs occurrence [19], and our results showed that chemotherapy was a risk factor for TEEs recurrence as well.

In the univariate analysis, our study found that LDH and D-dimer may be relevant to TEEs occurrence in ROS1 rearranged NSCLC. And the Fine and Gray test showed that elevated D-dimer may be related to the risk of TEEs. The increased TEEs risk among these patients indicated that ROS1 rearranged patients were at a hypercoagulable state. And D-dimer can reflect the activation of the hemostatic system, which demonstrated that a high-level D-dimer might associate with thrombosis [10, 20]. Repeat assessment indicated that D-dimer has a considerable TEEs predictive capacity among cancer patients [20–22]. This study suggested that oncogenic status was closely associated with the hypercoagulable profile including ROS1 rearrangement.

Patients with ROS1 rearrangement were treated with ROS1 TKIs as preferred, such as crizotinib in the first-line, and ceritinib after progression. Kaplan-Meier survival analysis showed that among patients receiving ROS1 TKIs in the first-line therapy (23 of 46, 50%), patients without TEEs had better median PFS than those with TEEs patients, which was consistent with previous studies [23]. The OS among patients with TEEs tended to be shorter than those without TEEs. Nevertheless, there was no statistical significance.

Our study had some limitations. First, due to the retrospective nature of the cohort, there was recall bias in our study. Although we included multicenter data, the sample size was relatively small, because ROS1 rearrangement was a rare variation. And the regression analysis without correction for multiple comparisons could influence the stability of the results. These results must be further validated by a large-scale prospective study. Finally, the lack of TEEs screening in our study may result in the underestimation of the incidence of TEEs among the enrolled patients since asymptomatic TEEs may be missed.

## Conclusion

Our study was a Chinese multicenter study that reported a high incidence rate of TEEs occurrence and TEEs recurrence in patients diagnosed with ROS1 rearranged advanced NSCLC. A high level D-dimer suggested that ROS1 rearranged NSCLC patients were at a hypercoagulable state, and chemotherapy tended to be the risk factor for TEEs recurrence. And the response to ROS1 TKIs was greater in patients without TEEs. Further large-scale prospective studies are needed to confirm these findings.

## Data Availability

Data were collected from all the patients. The datasets used and analyzed during the current study are available from the corresponding author on reasonable request.

## Abbreviations

NSCLC: Non-small cell lung cancer
TEE: Thromboembolic event
OS: Overall survival
PE: Pulmonary embolism
PFS: Progression free survival
TKI: Tyrosine kinase inhibitor
EGFR: Epidermal growth factor receptor
ALK: Anaplastic lymphoma kinase
FISH: Fluorescence in situ hybridization
IHC: Immunohistochemistry
RT-PCR: Reverse transcription-polymerase chain reaction
NGS: Next generation sequencing
ATE: Arterial thromboembolism
VTE: Venous thromboembolism
DVT: Deep vein thrombosis
CT: Computed tomography
MRI: Magnetic resonance imaging
CEA: Carcinoembryonic antigen
LDH: Lactate dehydrogenase
CRP: C-reactive protein
CCI: Charlson Comorbidity Index
KM: Kaplan Meier
RECIST 1.1: Response Evaluation Criteria In Solid Tumors version 1.1
PS: Performance status
LMWH: Low molecular weight heparin
